# Influence of the Prepolymer Type and Synthesis Parameters on Self-Healing Anticorrosion Properties of Composite Coatings Containing Isophorone Diisocyanate-Loaded Polyurethane Microcapsules

**DOI:** 10.3390/polym13050840

**Published:** 2021-03-09

**Authors:** Matic Šobak, Danaja Štular, Žiga Štirn, Gregor Žitko, Nataša Čelan Korošin, Ivan Jerman

**Affiliations:** 1 National Institute of Chemistry, Hajdrihova 19, 1000 Ljubljana, Slovenia; matic.sobak@ki.si (M.Š.); danaja.stular@ki.si (D.Š.); ziga.stirn@ki.si (Ž.Š.); gregor.zitko@ki.si (G.Ž.); 2 Jožef Stefan International Postgraduate School, Jamova 39, 1000 Ljubljana, Slovenia; 3 Faculty of Chemistry and Chemical Technology, University of Ljubljana, Večna pot 113, 1001 Ljubljana, Slovenia; natasa.celan@fkkt.uni-lj.si

**Keywords:** isocyanate prepolymers, polyurethane microcapsules, isophorone diisocyanate, composite coatings, anticorrosion coatings, self-healing

## Abstract

Self-healing anticorrosion composite coatings containing isophorone diisocyanate-loaded polyurethane microcapsules were developed, and comprehensive research on prepolymer and microcapsules synthesis, as well as functional composite coatings preparation and characterization, was performed. The influence of the prepolymer type and the concentration of the stabilizing agent used in the synthesis procedure on the properties of the microcapsules was studied in detail. For this purpose, three different prepolymers were prepared from toluene-2,4-diisocyanate (TDI) and either glycerol, 1,4-butanediol, or 1,6-hexanediol, and their chemical properties were investigated. Microcapsules were synthesized from the obtained prepolymers, according to the oil-in-water polymerization method, where 1,6-hexanediol was used as a chain extender, while the concentration of the stabilizing agent in the synthesis procedure was varied. Microcapsules prepared from TDI-glycerol prepolymer, synthesized in the presence of 10 wt% of the stabilizing agent, showed superior chemical, morphological, and thermo-gravimetrical properties; thus, they were incorporated into the coating in the concentration of 20 wt%. The prepared composite coatings demonstrated self-healing and anticorrosion properties, and thus the developed microcapsules show great potential for the incorporation into the composite anticorrosion coatings at critical points where damage can easily occur, providing longer and more efficient anticorrosion protection.

## 1. Introduction

According to the World Corrosion Organization, the cost of the corrosion of different metallic surfaces is estimated to be 1.3–1.4 trillion euros annually, representing 3.1–3.5% of the gross domestic product [1]. In the past, toxic and carcinogenic materials were used as corrosion-protective metal coatings, and thus the need for less toxic materials with the same protective properties, durability, long-term performance, and cost-effectiveness is on the rise [2,3]. Polymers which are prone to scratching and can efficiently heal themselves autonomously are of great interest since they can be used for the development of materials with self-healing properties. Among the latter, microcapsules show great potential for mass production, since they are easy to prepare and can be easily dispersed in the coating material matrix [4,5]. Encapsulated self-healing materials can be released from the microcapsules upon the mechanical damage of the coating, where they can react with a catalyst which is present in the coating, other microcapsules, or they can react with the oxygen or moisture from the environment. In this manner, a film is formed, which fills the damaged area, protecting the material from water and other oxidizing species that could cause electrochemical reactions and provoke corrosion of the material [6,7,8].

Isophorone diisocyanate (IPDI) is a widely used functional self-healing material which possesses the ability to react with water efficiently. Upon contact of IPDI with water or water vapor present in the environment, isocyanate groups transform into amino groups, which have the ability to react with isocyanate and urethane groups present in the system matrix, forming polymeric materials such as polyureas and allophanates, which could provide the anticorrosion protection of the materials [9]. Since water is required for the initiation of the corrosion processes, the use of a catalyst to initiate the self-healing process is not necessary [10]. Furthermore, among the microcapsule shell wall materials, polyurethane (PU) is of great interest considering that the majority of the studied microcapsule shell wall materials contain toxic and carcinogenic formaldehyde [11], and thus PU is a promising formaldehyde-free eco-friendly material that is safe to use [12,13].

IPDI-loaded PU microcapsules were first developed by Yang et al. [14], and continue to be investigated to this day [10,15,16,17,18,19,20,21,22,23,24]. The PU microcapsule shell wall is formed upon the reaction between the polyisocyanate prepolymers and the alcohol or hydroxyl-based chain extender. The important parameters influencing the quality of the shell wall are the length of the prepolymer molecular chain, its degree of branching, and its chemical composition. Among those, the amount of the isocyanate (NCO) groups in the prepolymer is of great importance, thus even though microcapsules could be prepared from the commercially available polyisocyanate prepolymers [10,15,21], researchers are focusing on the synthesis of isocyanate prepolymers with a higher content (~20 wt%) of NCO groups, which allow the formation of a firm PU microcapsules shell wall. In this manner, the PU microcapsules loaded with IPDI were formed with the use of toluene 2,4-diisocyanate (TDI) prepolymer and 1,4-butanediol as a chain extender [14,18,19]. In another study, the prepolymer was prepared from TDI and 2-ethyl-2-hydroxy methyl-1,3-propanediol, and the microcapsules were formed with the use of dibutyltin dilaurate as a reaction catalyst and 1,4-butanediol as a chain extender [17]. Sondari et al. formed PU microcapsules by using TDI and glycerol, where glycerol was used as a chain extender [23], while Kardar [16] used TDI and either 1,4-butanediol, 1,6-hexanediol, or glycerol, where 1,4-butanediol, 1,6-hexanediol, and glycerol were used as both polyols and chain extenders. More recently, diphenyl methane diisocyanate (MDI) was used for PU microcapsule shell wall formation for the encapsulation of IPDI. The shell wall synthesis was formed by the reaction of prepolymer with a polyol (1,4-butanediol) [21]. MDI was also used for the preparation of PU/polyurea or PU/polyurea-silica hybrid shell microcapsules for the encapsulation of IPDI [22], or methylenediphenyl diisocyanate (MDI) [24]. In this case, microcapsules were prepared from commercially available MDI in combination with different H sources. The concentration of the NCO groups in MDI stands at 31 wt%, and due to the higher reactivity of MDI in comparison to IPDI and therefore better shell-forming ability, the encapsulation yield IPDI increased.

Nevertheless, comprehensive research, including the synthesis of different TDI prepolymers, preparation of PU microcapsules, and their inclusion into the coatings, has not yet been performed. In this study, the influence of the prepolymer type on the chemical and morphological properties of microcapsules, as well as the protective and self-healing properties of the composite coatings, were researched. For this purpose, three different prepolymers were synthesized and studied, namely TDI-glycerol, TDI-1,4-butanediol, and TDI-1,6-hexanediol. The prepolymers were used for the synthesis of PU microcapsules loaded with IPDI, prepared according to oil-in-water synthesis with the use of 1,6-hexanediol as a chain extender. The influence of different concentrations of stabilizer in the water phase on the morphological and chemical properties of the microcapsules was also studied, by using 7, 10, and 17 wt% concentrations of gum arabic (GA). Selected microcapsules with the most promising morphological and chemical properties were incorporated into a mixture of commercially available coatings in the concentration of 20 wt%, and the self-healing and anticorrosion properties of the composite coatings were investigated.

## 2. Materials and Methods

### 2.1. Materials

Toluene 2,4-diisocyanate (TDI) (Sigma Aldrich, St. Louis, MO, USA), glycerol (Sigma Aldrich, St. Louis, MO, USA), 1,4-butanediol (Merck, Darmstadt, Germany), 1,6-hexanediol (Merck, Darmstadt, Germany), and chlorobenzene (Merck, Darmstadt, Germany) were used for the preparation of TDI-prepolymers, and cyclohexanone (ABCR, Karlsruhe, Germany) was used as a solvent. For the microcapsule synthesis, isophorone diisocyanate (IPDI) (Sigma Aldrich, St. Louis, MO, USA) was used as a core material, gum arabic (GA) (Sigma Aldrich, St. Louis, MO, USA) was used as a surfactant and cyclohexanone (ABCR, Karlsruhe, Germany), was used as a solvent. For the coating’s preparation, polyol (LUMIFLON LF9716 fluoropolymer consisting of alternating fluoroethylene and vinyl ether segments with an OH value of 170 KOH/g, AGC Chemicals, Exton, PA, USA) and diisocyanate (Desmodur N3900 aliphatic isocyanate resin based on hexamethylene diisocyanate, Convestro, Leverkusen, Germany) were used, and 2,2-dimethoxypropane (Sigma Aldrich, St. Louis, MO, USA) was used as a thinner.

### 2.2. Sample Preparation

#### 2.2.1. Synthesis of TDI-Prepolymers

**Figure 1:**
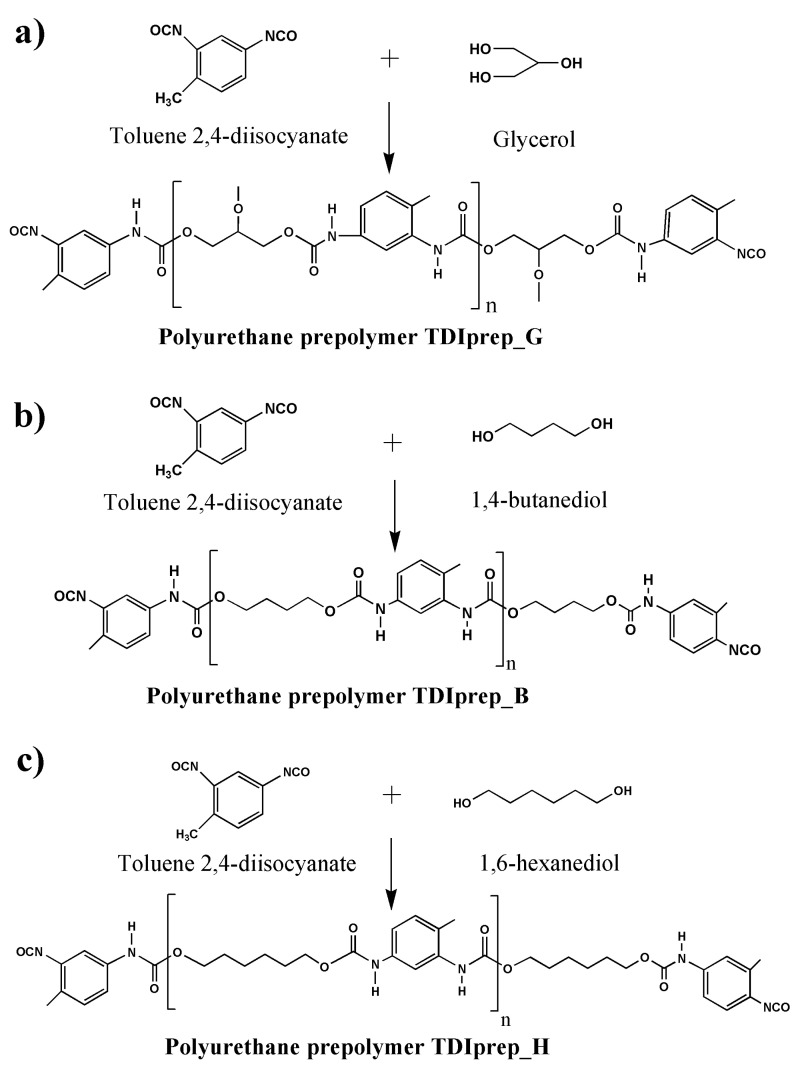
Schematic presentation of prepolymer synthesis, using toluene 2,4-diisocyanate and glycerol (**a**), 1,4-butanediol (**b**), or 1,6-hexanediol (**c**).

For the preparation of TDI-prepolymers, 20.90 g (120 mmol) of toluene 2,4-diisocyanate (TDI) was dissolved in 40 mL of dried cyclohexanone. The reaction vessel was purged with dried nitrogen to prevent any secondary hydrolysis. The temperature of the mixture was gradually raised to 80 °C with a heating rate of 2 °C/min and was continuously purged with nitrogen. When the temperature reached 80 °C, either 3.68 g (40 mmol) of glycerol, 5.41 g (60 mmol) of 1,4-butanediol, or 7.10 g (60 mmol) of 1,6-hexanediol was added into the synthesis mixture, and after the addition all having identical 100% excess of NCO groups. The reaction was left to stir at 80 °C for 24 hours. After 24 h, the resulting reaction mixture was evacuated at 25 mBar and 95 °C for 8 h in order to remove the unreacted isocyanate and cyclohexanone. The synthesis reaction for each prepolymer is schematically presented in Figure 1.

#### 2.2.2. Synthesis of IPDI-Loaded Polyurethane Microcapsules

**Figure 2:**
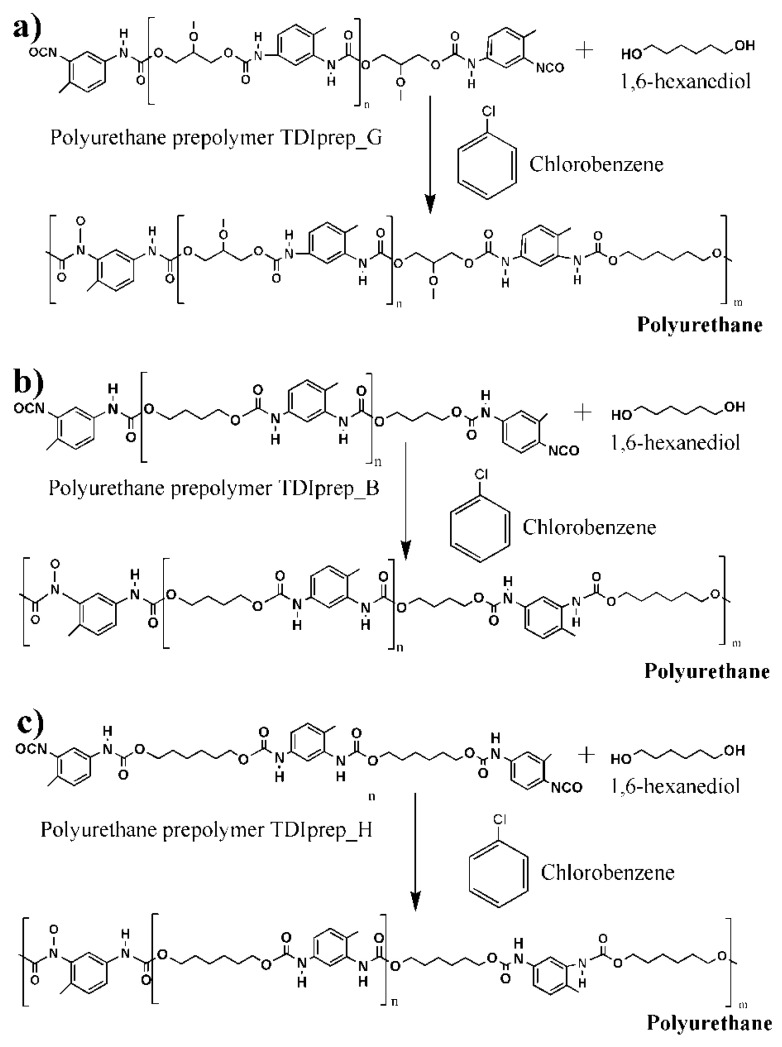
Schematic presentation of polyurethane microcapsule shell wall synthesis, using 1,6-hexanediol as chain extender and prepolymers toluene 2,4-diisocyanate–glycerol (**a**), toluene 2,4-diisocyanate-1,4-butanediol (**b**), and toluene 2,4-diisocyanate-1,6-hexanediol (**c**).

For the preparation of the water phase, 4,5 g of chain extender 1,6-hexanediol and GA in concentrations of 7, 10, or 17 wt% were added in 30 mL of Mili-Q water and left to stir overnight at room temperature. Oil phases were prepared by dissolving each of the TDI-prepolymers—TDI-glycerol, TDI-hexanediol, or TDI-butanediol (2.9 g)—in 4 g of chlorobenzene at 70 °C, followed by the addition of 9.5 g of IPDI. The water phase was warmed up to 70 °C at constant stirring (600 rpm), and the oil phase was added dropwise. The microcapsules were left to form for 1 h at 70 °C during constant stirring at 600 rpm. After the synthesis, deionized water was added to the mixture, the resulting product was allowed to settle on the bottom of the flask, the organic phase was decanted, fresh deionized water was added, and the resulting product was filtered and washed with plenty of water. The obtained capsules were air-dried for 24 h. The formation of a polyurethane shell from each of the three prepolymers is schematically presented in Figure 2.

#### 2.2.3. Preparation of the Composite Coatings

**Figure 3:**
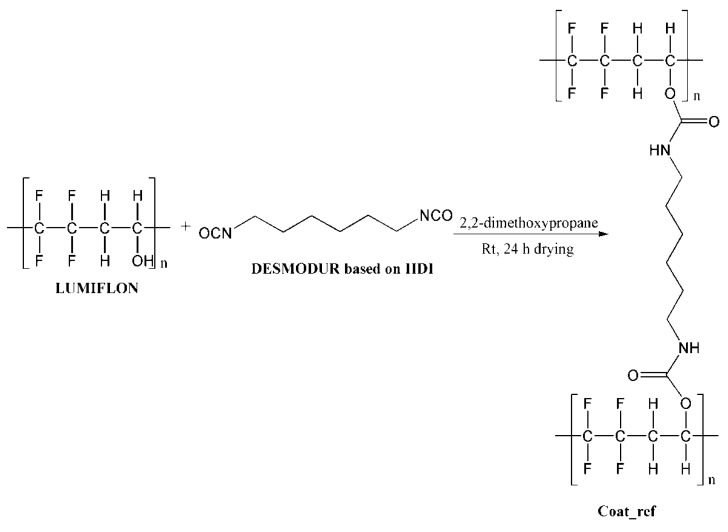
Schematic presentation of chemical reaction during coating preparation.

For the preparation of the composite coatings, selected microcapsules (20 wt%) were mixed in the mixture of Lumiflon LF 9716 (8.5 g) and Desmodur N3900 (2.12 g). As a thinner, 3.65 g of 2,2-dimethoxypropane was used. Coatings were applied on standard metal testing plates using a dip-coating procedure with ~30 µm thickness, and left to dry overnight. For the purpose of comparison, coating without the microcapsules was also applied on metal plates under the same conditions, where the thickness of the coating was ~25 µm. The completion of the curing reaction of the coating was confirmed by FT-IR analysis, by monitoring the disappearance of the NCO peak at 2276 cm^−1^. The chemical reaction which occurred during the coating preparation is shown in Figure 3.

**Figure 4:**
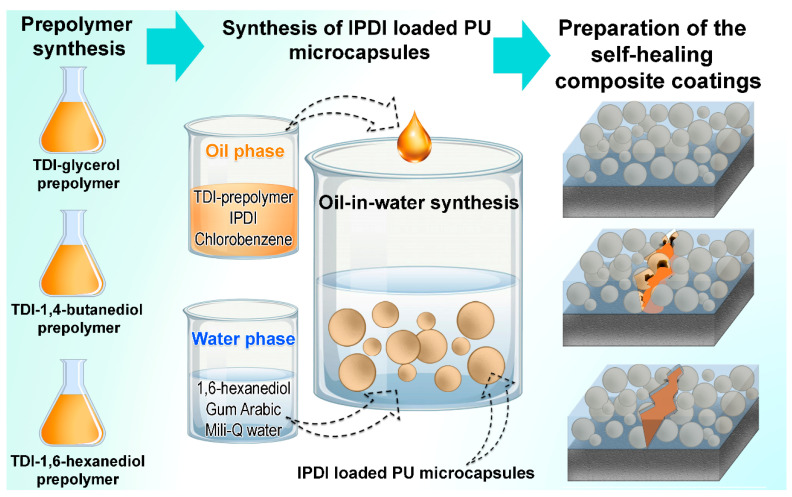
Schematic presentation of the sample preparation process.

Schematic overview of the process of preparation of the samples is depicted in Figure 4 and the corresponding sample abbreviations with material descriptions are presented in Table 1.

### 2.3. Analysis and Measurements

#### 2.3.1. Chemical Properties of the Synthesized Materials

FTIR spectra of prepared materials were obtained on Bruker (Vertex 70v, Billerica, MA, USA). The spectra were recorded over the range of 4000–600 cm^−1^ with a resolution of 4 cm^−1^ and averaged over 32 spectra. In the case of the microcapsule analysis, the studied microcapsules were frozen with liquid N_2_ and crushed, followed by washing of the product with acetone and filtering. The FTIR spectra were taken for the crushed microcapsules, the filtered material, and IPDI alone for the purpose of comparison.

Free NCO content in the prepared prepolymers was calculated by titration. For this purpose, 3 g of the synthesized prepolymers were weighed in 250 mL of the pre-dried flask, and 20 mL of dry toluene was added to dissolve the sample. After the sample had dissolved, 20 mL of freshly prepared 2 M di-n-butylamine was added to the solution. The mixture was allowed to mix for 20 min with vigorous stirring, followed by the addition of 100 mL of isopropanol and 1 mL of 0.04 wt% solution of bromophenol blue. The product was mixed and titrated with standardized 1 M aqueous hydrochloric acid until the colour of the solution turned yellow. The same procedure was performed on the blank. The isocyanate equivalent (I.E.) was calculated according to Equation (1):

(1) $$NCO,\;\%=\frac{\left[\left(B-V\right)\times M\times 0.0420\right]}{W}\;\times \;100$$

where *B* represents the volume of HCl for titration of the blank, *V* represents the volume of HCl used for titration the prepolymer, *M* represents the molarity of HCl, and *W* represents the mass of prepolymer in grams.

^1^H and ^13^C NMR spectra were recorded on a Bruker 600 MHz NMR spectrometer, model Avance Neo 600 (Fällanden, Switzerland). Samples were prepared in deuterated acetone, C_3_D_6_O, with tetramethylsilane (TMS) as an internal standard (δ(TMS) = 0.0 ppm). 

#### 2.3.2. Thermogravimetric Properties of the Materials

Differential scanning calorimetric (DSC) and thermogravimetric (TGA) analyses were performed on the synthesized prepolymers and microcapsules in order to study their reactivity and thermal stability utilizing a Mettler Toledo (Schwerzenbach, Switzerland) DSC 1 Analyzer and simultaneous TGA/DSC1 Thermogravimetric Analyser. The DSC analysis of prepolymers was performed in 40 µL aluminium crucibles filled with 2.10 to 2.83 mg of each sample and sealed with a pierced lid. After 15 min of isothermal conditioning in a nitrogen atmosphere at a flow rate of 30 mL/min at 25 °C, the samples were dynamically heated up to 450 °C, at a heating rate of 10 K/min. The TGA measurements were performed in 150 µL open platinum crucible heating from 25 to 800 °C at 10 K/min in a nitrogen atmosphere at a flow rate of 50 mL/min, including initial isothermal conditioning at room temperature. The sample masses varied between 6.80 and 7.47 mg. The baseline measured with an empty platinum crucible was subtracted for all the measurements. For the temperature decomposition interval of prepared prepolymer materials and the microcapsules and to determine different decomposition rates, the 1st derivative thermogravimetric (DTG) curves were obtained.

#### 2.3.3. Morphological Properties of the Materials

The morphology and size of the synthesized microcapsules and prepared coatings were observed using a scanning electron microscope (SEM, Zeiss Supra 35VP, Carl Zeis AG, Jena, Germany) with an accelerating voltage of 1.0 kV. All of the microcapsules were applied to a conductive carbon tape and coated with approximately 6 nm thick conductive platinum layer in order to prevent electrical discharge.

The average size of the synthesized microcapsules was determined from the SEM images, using ImageJ software [25]. The gathered values represent an average of approximately 200 measurements, although due to the large size of some microcapsules, a minimum of 50 measurements was performed and size distribution graphs were prepared.

#### 2.3.4. Determination of Self-Healing and Anticorrosion Properties of the Coatings

With the use of a razor blade, ~100 µm wide scratches were made in the coatings. Damage on the samples was observed using Leica EZ4W optical microscope (Leica, Wetzlar, Germany). For determining the anticorrosion performance of the coatings, the scratched samples were immersed into 3.5 wt% of NaCl water solution for 3 h. Afterwards, the samples were once again studied with an optical microscope. Additionally, samples with incorporated microcapsules were also observed under SEM, under conditions described in Section 2.3.3.

## 3. Results and Discussion

### 3.1. Characterization of Prepolymers

**Figure 5:**
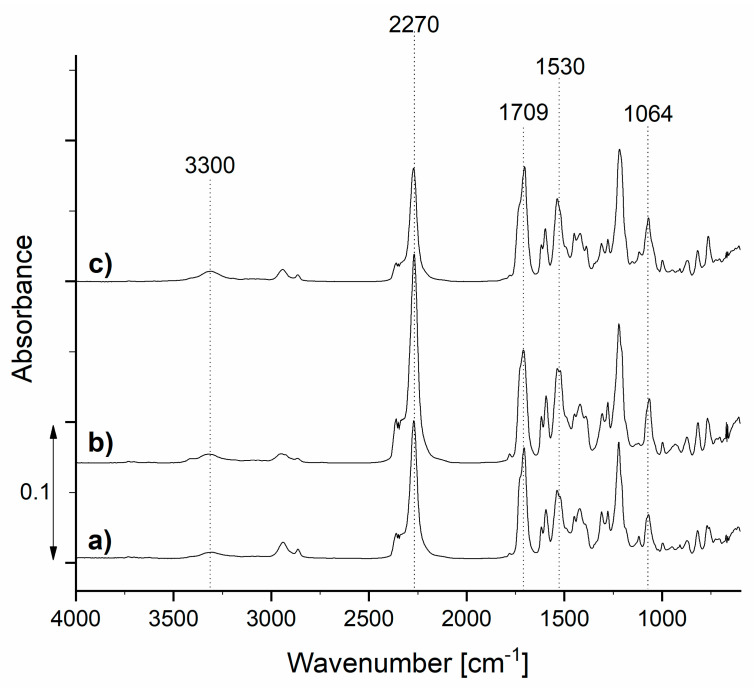
FTIR spectra of the synthesized prepolymers TDIprep_G (**a**), TDIprep_H (**b**), and TDIprep_B (**c**).

In the first step of the research, the prepolymers TDIprep_G, TDIprep_B, and TDIprep_H were synthesized. All of the obtained prepolymers appeared as highly viscous yellow resin-like products. In order to study their chemical composition, FTIR analysis was performed, and the results are depicted in Figure 5.

Prepared TDI prepolymers show absorption bands at about 3300 and 1530 cm^−1^, belonging to the bonded N–H stretching and N–H bending of the polyurethane group, respectively. The peaks in the area from 3000 to 2840 cm^−1^ belong to the C-H stretching vibrations, the absorption band at 1064 cm^−1^ can be attributed to C–O–C ester bonds (1000–1200 cm^−1^), and the absorption band at 1709 cm^−1^ represents the carbonyl (–CO) group of polyurethane (1870–1650 cm^−1^). These results clearly show the presence of polyurethane groups, as well as isocyanate groups in all three samples, which confirmed successful isocyanate prepolymer synthesis. Moreover, the absorption band at 2270 cm^−1^ corresponds to the stretching vibration of the isocyanates (–NCO), meaning that the unreacted isocyanate groups, which are crucial for the reaction with the chain extender and consequently the formation of the polyurethane microcapsule shell, were still present at the terminal end of the polymer chain [16,23,26,27].

The exact concentration of the NCO groups was also determined by titration, and the results show the lowest NCO content for the prepolymer TDIprep_G, where 19.20 wt% concentration was present. Prepolymer TDIprep_B contained 20.50 wt%, and the prepolymer TDIprep_H showed the highest NCO, with a 21.10 wt% concentration. According to the literature, the concentration of NCO of about 20 wt% is sufficient for the formation of the shell wall of the PU microcapsules and is higher than the NCO content of the commercial polymers [14,15]. 

Additionally, ^1^H NMR studies of prepolymers were performed, and the results are presented in supporting material (Figure S1). In all three prepolymers, amide, aromatic, and aliphatic protons were confirmed. Therefore, ^1^H NMR studies were in agreement with IR analysis performed, both confirming the polymeric structure of isocyanates.

**Figure 6:**
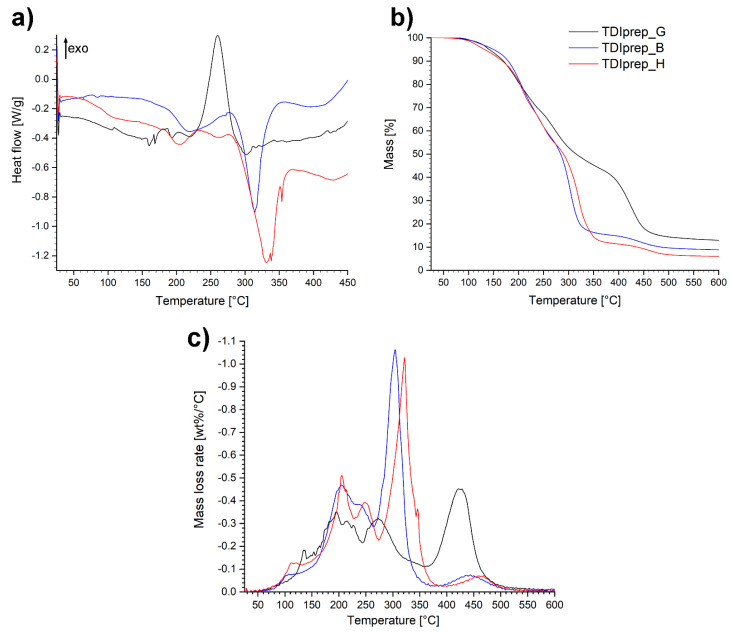
DSC (**a**)**,** TGA (**b**) and DTG (**c**) thermoanalytical curves of studied toluene-2,4-diisocyanate (TDI)-prepolymers in a nitrogen atmosphere.

Furthermore, in order to obtain thermal properties of prepared prepolymer materials, DSC and TGA analyses were performed (Figure 6).

From the DSC diagrams of the prepolymer samples (Figure 6a), endothermic and exothermic peaks in the area between ~150 and ~280 °C were observed. A significant exothermic peak was present in the case of TDIprep_G, while in the case of prepolymers incorporating 1,4-butanediol and 1,6-hexanediol, only minor endothermic events with peaks at 220 and 205 °C, respectively, were observed. Further on, at the onset temperature at 290 °C, both linear polymers TDIprep_B and TDIprep_H displayed considerable endothermic effect with peaks at 315 and 330 °C, respectively, while the mentioned effect was not present in the case of TDIprep_G prepolymer. The difference between the spectra of TDIprep_G and both linear prepolymers generated two major questions that need further clarification. Firstly, the reason why TDIprep_G displayed such immense exothermic effect in the 230–300 °C range compared to the other two linear prepolymers, and secondly, the reason for the absence of endothermic effect observed above 300 °C in case of TDIprep_G, while it is a dominant signal on DSC curves of TDIprep_B and TDIprep_H.

The first postulation was that a certain degree of free OH groups was present in the molecule of the glycerol-based prepolymer, which could react with NCO groups only at an elevated temperature due to steric constraints in the molecule. The formation of additional crosslinks at high temperatures (250 °C) would then postpone the degradation process and hence evaporation of the starting materials would not be as evident as in the case of linear prepolymers (TDIprep_B and TDIprep_H). Nevertheless, this theory was dismissed by IR and CAN (ceric ammonium nitrate) colour test, which showed that free OH groups were not present in the molecule. Additionally, DSC analysis of TDIprep_G, was performed in a nitrogen atmosphere up to 600 °C (Figure S2). Based on the results, there was no endothermic peak present above 300 °C; instead, two additional exothermic peaks were confirmed. This furtherly invalidated our initial postulation. 

The remaining explanation would be that the exothermic event in the case of TDIprep_G was an indication of a chemistry change. This phenomenon would also explain the absent endothermic peak above 300 °C, and the appearance of two exothermic contributions at higher temperatures in the DSC spectra of TDIprep_G (Figure S2). Even though the difference in the degradation pattern is clear evidence of a chemistry change during excessive heating in the case of TDIprep_G prepolymer, the reason why this change only occurred for the prepolymer TDIprep_G and not for the other linear prepolymers remains unclear, since all three prepolymers contain identical starting functional groups. Apparently, the presence of glycerol contributes towards higher reactivity of possible reactions taking place at higher temperatures in polyurethane/isocyanate mixture [28,29,30]. Nevertheless, this explanation remains elusive and is beyond the scope of this work. Furthermore, the debated chemistry evolves at high degradation temperatures of the material, which are not relevant for the use of the coatings developed in this work.

From the TG and DTG curves (Figure 6b,c), it is evident that the decomposition of the prepolymers shows a multi-step-mass loss tendency. The decomposition step, which occurs in the temperature range of 150–280 °C was similar for all three prepolymers and represents the degradation of the so-called hard segment, i.e., urethane functionality, evident on the DTG chart as two peaks at 200 and 250 °C. Hence, according to the known mechanism, the prepolymers decomposed to TDI and the respective diols/triols, in this temperature range [30,31]. At higher temperatures where the degradation of the polyols representing the soft segments occurs, the evaporation of diols at 300 and 320 °C for TDIprep_B and TDIprep_H prepolymers, respectively, was observed. In the case of TDIprep_G, the degradation step occurred at 420 °C (Figure 6c). Based on observations, prepolymer TDIprep_G showed greater thermal stability compared to the prepolymers TDIprep_B and TDIprep_H, as mass loss proceeds at a slower pace in two related heat-releasing events (Figure S2). The main contributor to this behavior was a higher degree of polyurethane 3D structure, due to the higher OH functionality of the alcohol used. The completion of the last decomposition step of prepolymers TDIprep_B and TDIprep_H at approximately 370 and 380 °C, respectively (Figure 6b), correlates to the temperature range of endothermic peaks observed on DSC curves. In comparison, the final degradation temperature of the prepolymer TDIprep_G occurs at approximately 510 °C, once again showing the better thermal stability of this prepolymer.

### 3.2. Characterization of the Microcapsules

**Figure 7:**
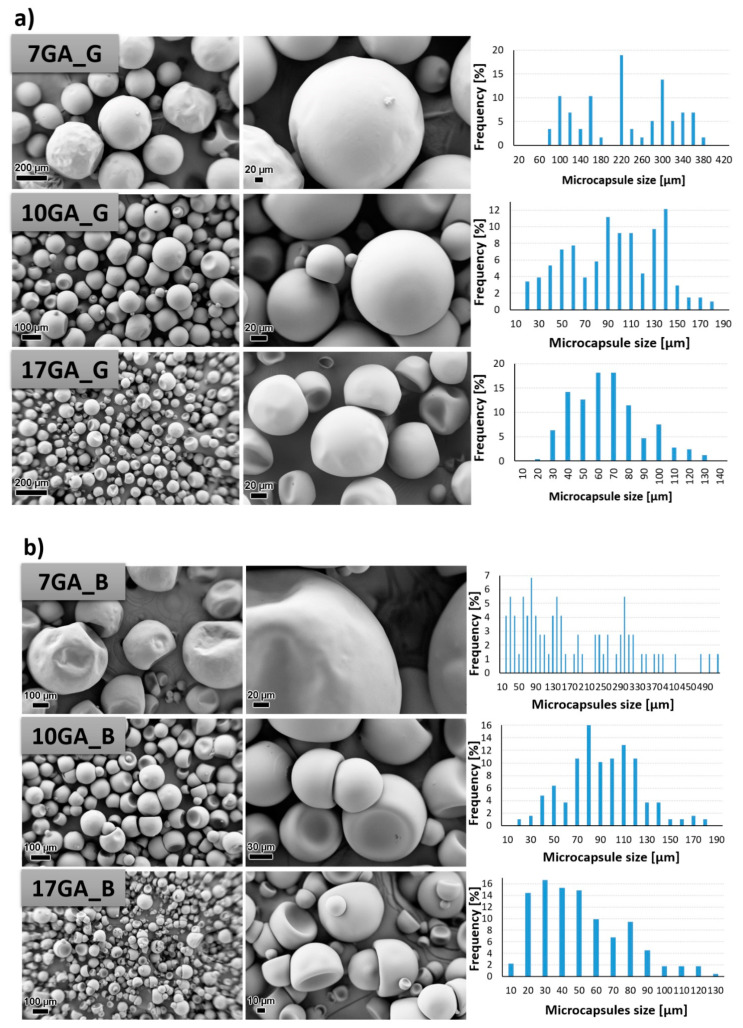

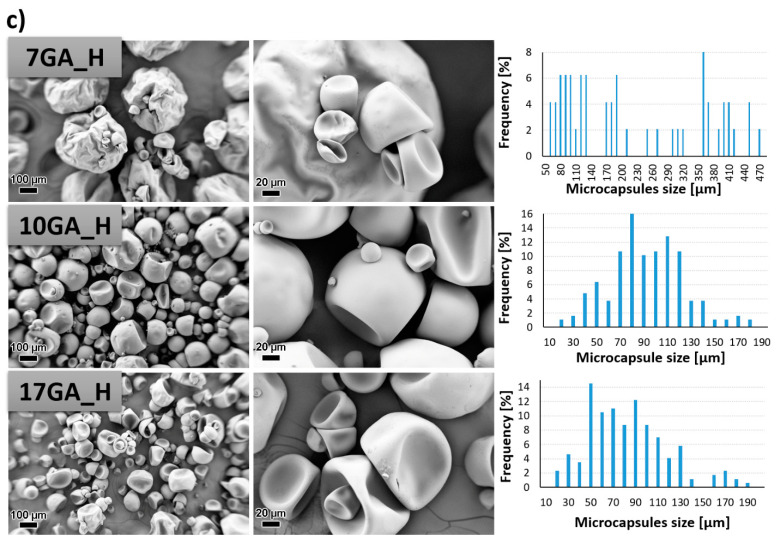
SEM micrographs taken under 200× (left column) and 1000× magnification (middle column) and size distribution of the prepared polyurethane (PU) microcapsules filled with isophorone diisocyanate (IPDI) (right column), prepared with various concentrations of gum arabic (GA) and prepolymer TDIprep_G (**a**), TDIprep_B (**b**) and TDIprep_H (**c**).

PU microcapsules loaded with IPDI were synthesized from the prepolymers using of 1,6-hexanediol as chain extender while the concentration of stabilizer GA in the water phase was varied in order to determine its influence on the morphological properties of the microcapsules. The morphology of the prepared microcapsules was investigated with the use of SEM, and the size of the microcapsules was determined using ImageJ software. The results are depicted in Figure 7.

PU microcapsules were formed during the synthesis procedure regardless of the used prepolymer or the concentration of the stabilizing agent. Overall, the shell wall of the microcapsules was non-porous, which was desirable so that the incorporated material cannot escape out of the microcapsule. Furthermore, it is evident that the chemical composition of the prepolymer used in the synthesis of the microcapsules greatly influenced the microcapsules’ morphology, and thus the most uniform morphology and the least deformed shape of the microcapsules was obtained when using prepolymer TDIprep_G (Figure 7a). Microcapsules synthesized with prepolymer TDIprep_B started to deform, and hence the shell was concaved and the round shape of the microcapsules was lost. The most deformed and concaved microcapsules were obtained when prepolymer TDIprep_H was used in the synthesis procedure. These results are consistent with the results obtained using DSC and TG (Figure 6), where superior reactivity and the thermal stability of the prepolymer TDIprep_G are shown. Moreover, greater cross-linking of the prepolymer TDIprep_G can be explained by the non-linear chemical composition of glycerol, and thus the polymer can be cross-linked in three dimensions, while 1,4-butanediol and 1,6-hexanediol are linear polymers, which limits their cross-linking density [23]. However, these results are not consistent with the results obtained by Kardar et al. [16], where the use of glycerol-based prepolymer led to the least favourable morphology and properties of the microcapsules. The reason for the inconsistency lies in the use of different chain extenders, thus in the literature, glycerol was used for prepolymer synthesis and as a chain extender, while in the present research, the glycerol-based prepolymer was combined with 1,6-hexanediol as a chain extender, which proved to be more suitable for the firm microcapsule shell wall formation from TDI-glycerol prepolymer. Furthermore, to the best of our knowledge, this is the first time that IPDI-loaded PU microcapsules with homogenous, non-wrinkled morphology were successfully obtained from the TDI-glycerol-based prepolymer.

The variation in the concentration of the stabilizer (GA) during the synthesis also significantly influenced the size and the morphology of the synthesized microcapsules. In general, the increase in the concentration of the GA decreased the size of the microcapsules, where the average diameter of the microcapsules 7GA_G, 10GA_G, and 17GA_G was 217.82, 89.79, and 61.14 µm, the microcapsules 7GA_B, 10GA_B, and 17GA_B had a diameter of 174.53, 89.33, and 45.28 µm, and the diameter of the microcapsules 7GA_H, 10GA_H, and 17GA_H measured 220.78, 95.44, and 77.73 µm, respectively. The lowest amount of the stabilizer used in the synthesis procedure also imposed the least uniform size distribution of the microcapsules, while the size distribution of the microcapsules synthesized with higher concentrations of 10 and 17 wt% GA was more uniform. When the lowest (7 wt%) concentration of GA was used in the synthesis, the shell wall of the prepared microcapsules became wrinkled, which was most obvious in the case of 7GA_H microcapsules. Hence, the microcapsules’ wrinkled morphology could be caused by the inconsistency in the reaction kinetics, elastic forces of the shell, and fluid influenced shear forces [16,18]. 

**Figure 8:**
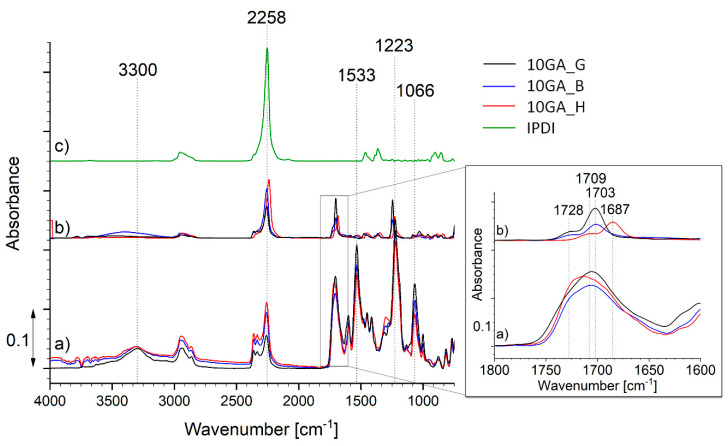
FTIR spectra of the crushed microcapsules 10GA_G, 10GA_B and 10GA_H (**spectrum a**), filtered core material (**spectrum b**), and pure IPDI (**spectrum c**).

The encapsulation of the IPDI inside the microcapsules shell walls was studied using FTIR (Figure 8). For that purpose, the microcapsules were frozen with N_2_, crushed, washed with acetone, and filtered. The FTIR spectra of the crushed microcapsules (spectrum a), filtered material (spectrum b) and neat IPDI (spectrum c) were recorded for the purpose of comparison. The spectra shown in Figure 8 belong to the samples 10GA_G, 10GA_B, and 10GA_H, while the spectra of other studied microcapsules are shown in Section S3 of Supporting Material.

The FTIR spectra of the crushed microcapsules (spectrum a) are in accordance with the spectra of the TDI-prepolymers presented in Figure 5, and thus they show absorption bands attributed to the formation of urethane linkage, namely the C–O–C bond (1000–1200 cm^−1^), the –OC group (1870–1650 cm^−1^), the absorption bands belonging to N–H stretching and N–H bending at about 3300 and 1530 cm^−1^, as well as the absorption spectra of the isocyanate groups (2280–2260 cm^−1^). The comparison of the IR spectrum of microcapsule shell (spectrum a) and core material (spectrum b) reveals that the shell matrix of the microcapsules is not intertwined with hydrogen bonds. This was evident from the spectra of the PU extracted from the core material of the microcapsule, which shows a double peak of the carbonyl group at 1728 and 1703 cm^−1^ (10GA_G and 10GA_B) or 1709 and 1687 cm^−1^ (10GA_H), representing hydrogen bonded carbonyl as well as carbonyl free groups, respectively. Additionally, the broadening and apparent blue shift from 3300 to 3408 cm^−1^ was observed in the case of N–H stretching vibration for the filtered core material of the sample 10GA_B [32,33]. When comparing the absorption bands of the crushed microcapsules to the spectra of microcapsule-filtered core material (spectrum b) and the spectra of pure IPDI (spectrum c), it is evident that some of the prepolymer residues can still be seen in the spectra of the filtered core material since the low-intensity absorption bands appear in the area between ~1100 and ~1700 cm^−1^, and in the case of the sample 10GA_H also at 3300 cm^−1^. Furthermore, the absorption band standing at 2270 cm^−1^, indicating the presence of the –NCO groups, was present in all of the studied spectra. Based on the spectral comparison, it is clearly evident that a major NCO peak can be allocated to the IPDI molecule. On the other hand, two peaks at higher wavenumber are observed in the case of crushed and extracted microcapsules (spectrum a and b). These peaks most probably belong to prepolymer or partially reacted prepolymer, which also became a part of the core material, after the shell formation. The reason for this phenomenon can be found in the excess of NCO groups compared to OH groups during the formation of the capsule shell.

**Figure 9:**
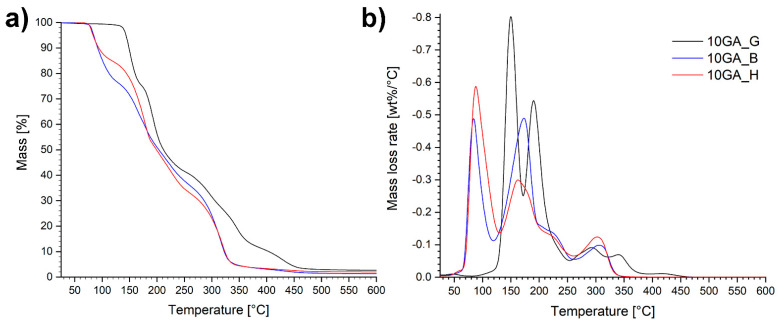
TGA curves in a nitrogen atmosphere (**a**) and corresponding DTG graphs (**b**) of the microcapsules 10GA_G, 10GA_B, and 10GA_H.

Thermogravimetric properties of the microcapsules 10GA_G, 10GA_B, and 10GA_H, determined using TGA and DTG analysis, are portrayed in Figure 9. 

The TGA and DTG plots of the studied microcapsules show that the analyzed materials are composed of various different phases with different decomposition rates, expressed as multiple-step-mass loss tendency. Overall, the microcapsules 10GA_G decomposed at higher temperatures compared to the microcapsules 10GA_B and 10GA_H, which confirms the greater crosslinking density of the polymer networks due to the three functional groups of glycerol, and therefore better thermal stability of the TDI-glycerol-based microcapsules. The first mass loss occurred at temperatures in the range of 80–130 °C for the samples 10GA_B and 10GA_H, and 130–180 °C for the sample 10GA_G, which was ascribed to the evaporation of the solvent chlorobenzene, the boiling point of which stands at 132 °C. Additionally, it was observed that during the first degradation step, sample 10GA_H loses the least amount of mass, i.e., approximately 15%, while on the other hand, 10GA_B and 10GA_G lost approximately 23%. This observed phenomenon is in accordance with the deformations and concaved structure of 10GA_H microcapsules, which cannot harbour the same amount of material as 10GA_B and 10GA_G microcapsules. The second mass loss step was attributed to the degradation of the urethane linkage in the microcapsule shell wall and the evaporation of IPDI, which was used as a functional core material. Furthermore, the third decomposition step could be ascribed to the elongated decomposition of the urethane linkage of the prepolymers to TDI and polyols, which represent the soft segments. The final mass loss stage of the studied microcapsules corresponds to the decomposition of the polyols determined with the TGA analysis (Figure 6b), and thus it indicates the decomposition of remaining soft segments with an observed shift in the thermal decomposition of TDI-glycerol-based microcapsules to higher temperatures.

### 3.3. Self-Healing and Anticorrosion Evaluation of the Coatings

**Figure 10:**
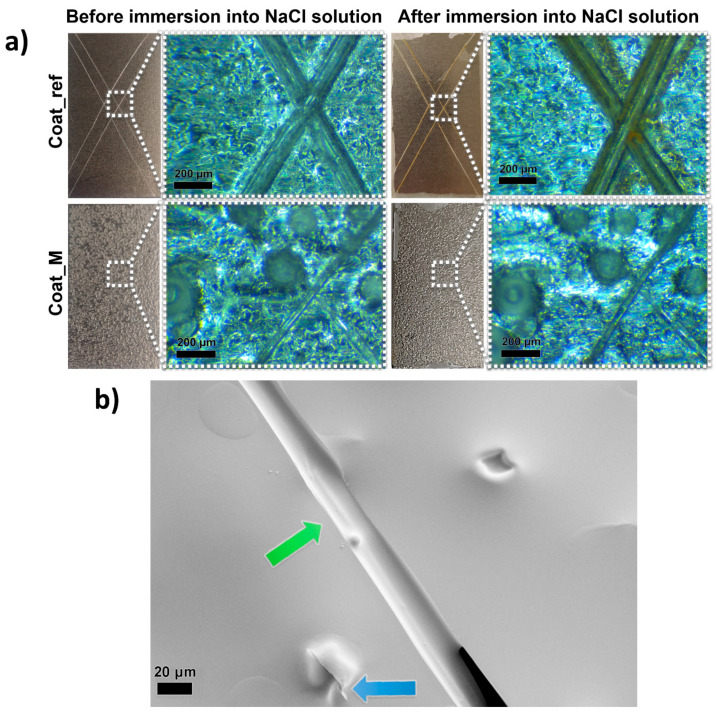
Visual presentation of the scratched samples Coat_ref and Coat_M taken with a camera and optical microscope, before and after the immersion of the samples into the NaCl solution (**a**) and SEM image of the scratched composite coating, showing the release of the IPDI from the ruptured microcapsule and filling of the scratched area with polyurethane film (**b**).

After the synthesis, the microcapsules 10GA_B and 10GA_H started to form larger agglomerates (photographs and SEM images are shown in Supporting Material Figure S4), which enabled their further use, and thus 10GA_G microcapsules were chosen for the incorporation into the anticorrosion self-healing coatings. The composite coatings containing 20 wt% of 10GA_G microcapsules were applied on standard metal testing plates. The SEM images of the applied composite coatings are presented in Figure S5. Next, the coatings were scratched with a razor blade in order to determine the self-healing ability. Damaged coating areas were studied using an optical microscope, before and after the immersion into the NaCl solution (Figure 10a).

When comparing scratched areas of the coatings Coat_ref and Coat_M, it is evident that the microcapsules incorporated into the composite coating greatly influenced the size of the scratched area. The scratches on the sample Coat_ref can be seen with the naked eye, while the scratches on the sample Coat_M can only be seen when observed under a microscope. Comparison of the two samples under an optical microscope reveals that the width of the scratch of the Coat_ref sample is approximately 100 µm wide, which is more than twice the width of the scratch made on the Coat_M sample, at approximately 40 µm. This can be explained by the release of the IPDI from the PU microcapsules upon the opening of the microcapsules, which filled the scratch and formed a PU film upon contact with water. The aforementioned PU film protected the metal plate substrates from corrosion, which can be seen from the photograph of the samples after the immersion in NaCl solution. Namely, no rust was detected after the immersion of the sample Coat_M into the NaCl solution, while the reference coated metal plate begun to rust on the damaged areas.

To gain further understanding of the self-healing mechanism of the composite coating, an SEM image of the scratched area of the sample Coat_M was taken, and the results are presented in Figure 10b. The self-healing performance imposed by the rupture of the IPDI-loaded microcapsules was observed, and thus the release of the IPDI from the ruptured microcapsule can be seen from the area marked with a blue arrow, while the green arrow marks the area where released IPDI filled the gap and formed the polyurethane film, resulting in the improved protective properties of the coating.

## 4. Conclusions

Anticorrosion self-healing composite coatings were developed with the use of isophorone diisocyanate (IPDI)-loaded polyurethane (PU) microcapsules and the influence of the prepolymer type, and the concentration of the stabilizing agent in the synthesis procedure on the self-healing anticorrosion properties of the coatings were studied. Compared to prepolymers synthesized from toluene 2,4-diisocyanate and 1,4-butanediol or 1,6-hexanediol, superior thermal stability and reactivity were determined for the prepolymer synthesized from toluene 2,4-diisocyanate and glycerol. Microcapsules filled with IPDI were prepared from the prepolymers and 1,6-hexanediol as a chain extender, where microcapsules prepared with glycerol-based prepolymer showed the most uniform morphology, the highest concentration of entrapped IPDI, and the greatest thermal stability among the microcapsules. With an increase in the stabilizing agent in the synthesis procedure, the size of the microcapsules decreased, and thus microcapsules synthesized with the use of 10 wt% of the stabilizing agent showed the most promising morphology and uniform size distribution. Microcapsules prepared from glycerol-based prepolymer in the presence of 10 wt% of the stabilizing agent were incorporated into the composite coatings in the concentration of 20 wt%. Prepared composite coatings showed excellent self-healing properties and anticorrosion protection of standard metal testing plates after scratching. The microcapsules developed in this study can be used in composite anticorrosion coatings at critical points where damage can easily occur, for long-lasting anticorrosion protection of metal surfaces.

## Figures and Tables

**Table 1:** Sample codes according to prepared materials.

Sample Abbreviations	Description of the Material
TDIprep_G	Prepolymer prepared from toluene 2,4-diisocyanate and glycerol
TDIprep_B	Prepolymer prepared from toluene 2,4-diisocyanate and 1,4-butanediol
TDIprep_H	Prepolymer prepared from toluene 2,4-diisocyanate and 1,6-hexanediol
xGA_G	Microcapsules loaded with isophorone diisocyanate, prepared from TDIprep_G, chain extender 1,6-hexanediol and various concentration of the stabilizer, GA, where variable x represents the wt% of GA (7, 10 or 17)
xGA_B	Microcapsules loaded with isophorone diisocyanate, prepared from TDIprep_B, chain extender 1,6-hexanediol and various concentration of the stabilizer, GA, where variable x represents the wt% of GA (7, 10 or 17)
xGA_H	Microcapsules loaded with isophorone diisocyanate, prepared from TDIprep_H, chain extender 1,6-hexanediol and various concentration of the stabilizer, GA, where variable x represents the wt% of GA (7, 10 or 17)
Coat_ref	Metal plates coated with coating reference without the microcapsules
Coat_M	Metal plates coated with composite coatings containing 20 wt% of microcapsules 10GA_G

## Data Availability

Data will be made available upon request.

## Supplementary Materials

The following are available online at https://www.mdpi.com/2073-4360/13/5/840/s1, Figure S1: ^1^H NMR of the studied prepolymers, Figure S2: DSC thermoanalytical curve of the TDIprep_G, Figure S3: FTIR spectra of the studied microcapsules synthesized with various prepolymers and stabilizing agent concentration, Figure S4: Photo and SEM images of microcapsules after the synthesis, Figure S5: SEM images of the composite anticorrosion coatings applied on metal plate.
