# Supplementary material for: Influence of the Prepolymer Type and Synthesis Parameters on Self-Healing Anticorrosion Properties of Composite Coatings Containing Isophorone Diisocyanate-Loaded Polyurethane Microcapsules
Source: Polymers (Basel). 2021 Mar 9;13(5):840. doi: 10.3390/polym13050840 (PMC7967154; doi:10.3390/polym13050840)
Supplement: Supplementary file 1 [file polymers-13-00840-s001.pdf]

polymers

MDPI

Supplementary Materials

Influence of the Prepolymer Type and Synthesis Parameters on Self-Healing Anticorrosion Properties of Composite Coatings Containing Isophorone Diisocyanate-Loaded Polyurethane Microcapsules

Matic Šobak [1,2], Danaja Štular [1], Žiga Štirn [1], Gregor Žitko [1], Nataša Čelan Korošin [3] and Ivan Jerman [1,*]

[1] National Institute of Chemistry, Hajdrihova 19, 1000 Ljubljana, Slovenia; matic.sobak@ki.si (M.**Š.**); danaja.stular@ki.si (D.**Š.**); ziga.stirn@ki.si (Ž.Š.); gregor.zitko@ki.si (G.Ž.)
[2] Jožef Stefan International Postgraduate School, Jamova 39, 1000 Ljubljana, Slovenia
[3] Faculty of Chemistry and Chemical Technology, University of Ljubljana, Večna pot 113, 1001 Ljubljana, Slovenia; natasa.celan@fkkt.uni-lj.si
* Correspondence: Ivan.Jerman@ki.si; Tel.: +386-1-4760-440

S1 ^{1}H NMR of the studied prepolymers

^{1}H NMR spectra of the studied prepolymers are presented in **Figure S1**. In all three prepolymers, amide, aromatic and aliphatic protons were confirmed. Therefore ^{1}H NMR studies were in agreement with IR analysis preformed, both confirming polymeric structure of isocyanates.

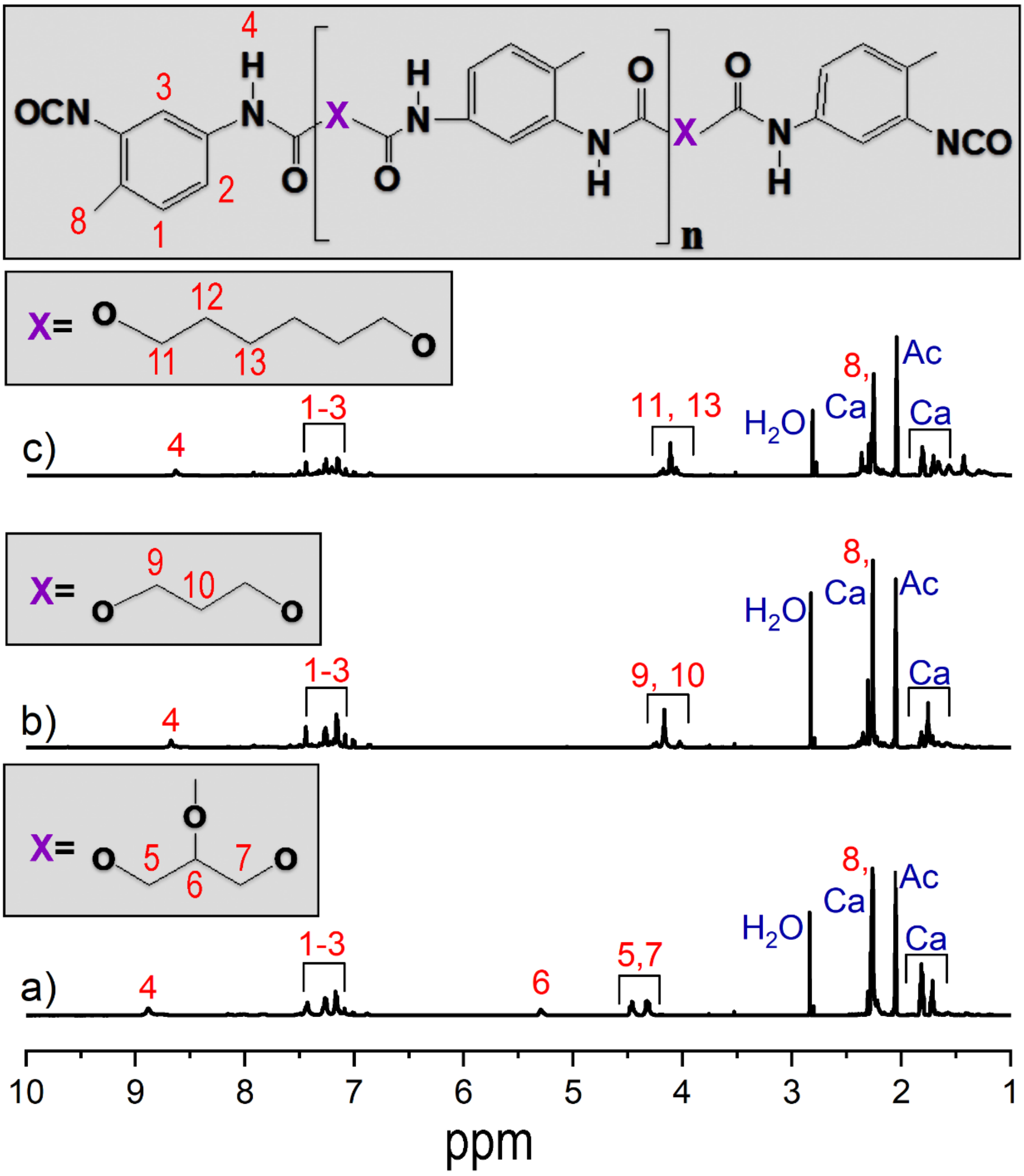

Figure S1. ^{1}H NMR spectra of the prepolymers TDIprep_G **(a)**, TDIprep_B **(b)** and TDIprep_H prepolymers **(c)**.

TDIprep_G: ^{1}H NMR: $(CD_3)_2CO$, δ (ppm)): 8.88 (s, 2H, NH), 7.43 (m, 2 H, Ar), 7.27 (m, 2H, Ar), 7.16 (m, 2H, Ar), 5,30 (s, end–O–$CH_2CH(O\text{-})CH_2$, 1H), 4.46 (m, end–O–$CH_2CH(O\text{–})CH_2$, 2H), 4.46 (m, end–O–$CH_2CH(OH)CH_2$, 2H), 2.26 (s, CH_3, 6H).

TDIprep_B: ^{1}H NMR $(CD_3)_2CO$, δ (ppm)): 8.68 (s, 1H, NH), 7.44 (m, 2 H, Ar), 7.26 (m, 2H, Ar), 7.16 (m, 2H, Ar), 4.17 (s, O–CH_2–CH_2, 4H), 2.26 (s, CH_3, 6H), 4.46 (m, end–O–$CH_2CH(OH)CH_2$, 2H), 2.26 (s, CH_3, 6H), 1.75 (m, O–CH_2–CH_2, 4H).

TDIprep_H: ^{1}H NMR $(CD_3)_2CO$, δ (ppm)): 8.64 (s, 1H, NH), 7.45 (m, 1 H, Ar), 7.26 (m, 1H, Ar), 7.16 (m, 1H, Ar), 4.22–4.05 (m, O–CH_2–CH_2, 4H), 2.26 (s, CH_3, 6H), 1.68–1.66 (m, aliphatic, 2H),1.59–1.55 (m, aliphatic, 2H),1.45–1.42 (m, aliphatic, 2H), 1.32–1.17 (m, aliphatic, 2H).

S2 DSC thermoanalytical curve of the TDIprep_G

DSC thermoanalytical curve of the prepolymer TDIprep_G recorded in a nitrogen atmosphere up to 600 °C is presented in **Figure S2**. No endothermic peaks occurred above 300 °C, while two additional exothermic peaks were present, indicating the chemistry change of the sample.

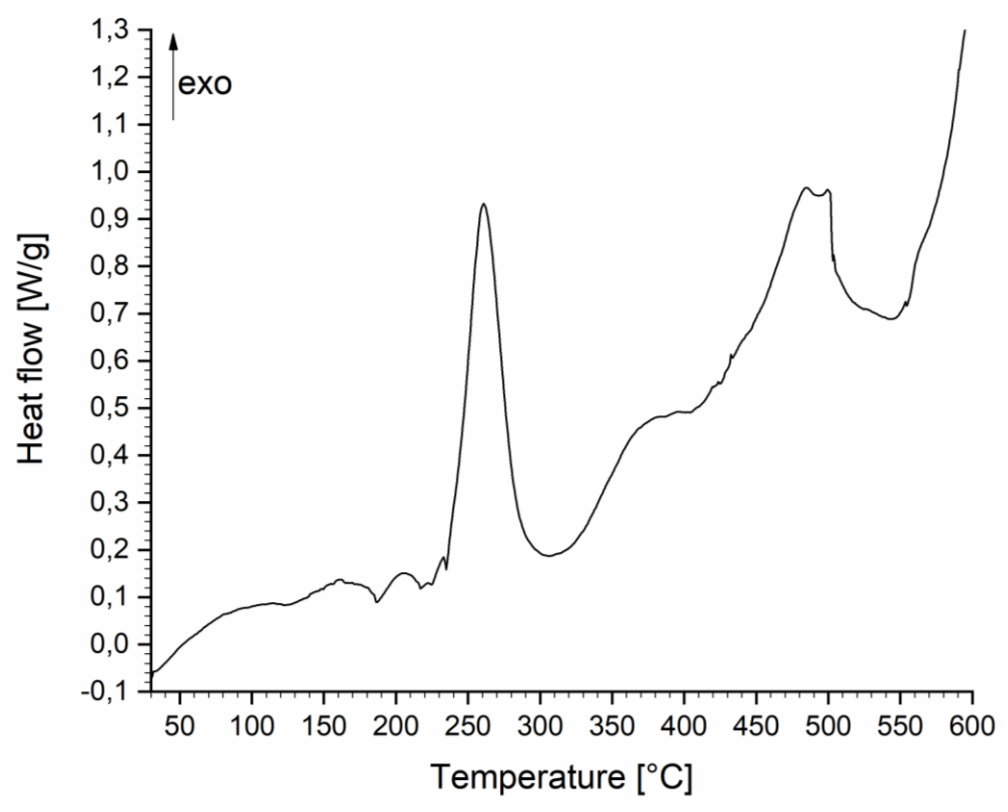

Figure S2. DSC thermoanalytical curve of the TDIprep_G in a nitrogen atmosphere.

S3 FTIR spectra of the studied microcapsules synthesized with various prepolymers and stabilizing agent concentration

The FTIR spectra of all of the synthesized microcapsules are presented in **Figure S3**. In specters A the measurements were performed on crushed microcapsules, specters B represent the filtered core material and specter C belongs to pure IPDI for the purpose of comparison. Comparison between specters A, B and C showed that some of the pre-polymer residues can still be seen in the spectra of filtered core material since the low intensity absorption bands appear in the area between ~1100 cm^{-1} and ~1700 cm^{-1}, as well as 3300 cm^{-1} in the case of the samples 7GA_G, 7GA_B and 10GA_B. No larger dissimilarities between the FTIR spectra of microcapsules prepared from different prepolymers can be detected.

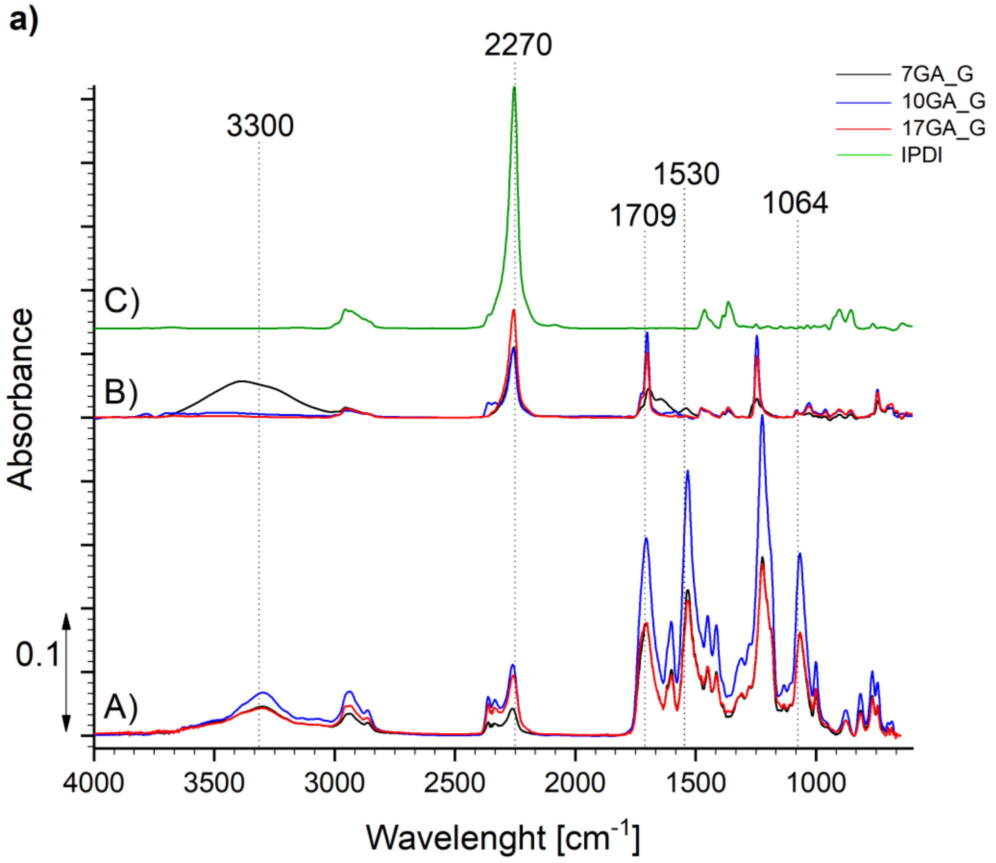

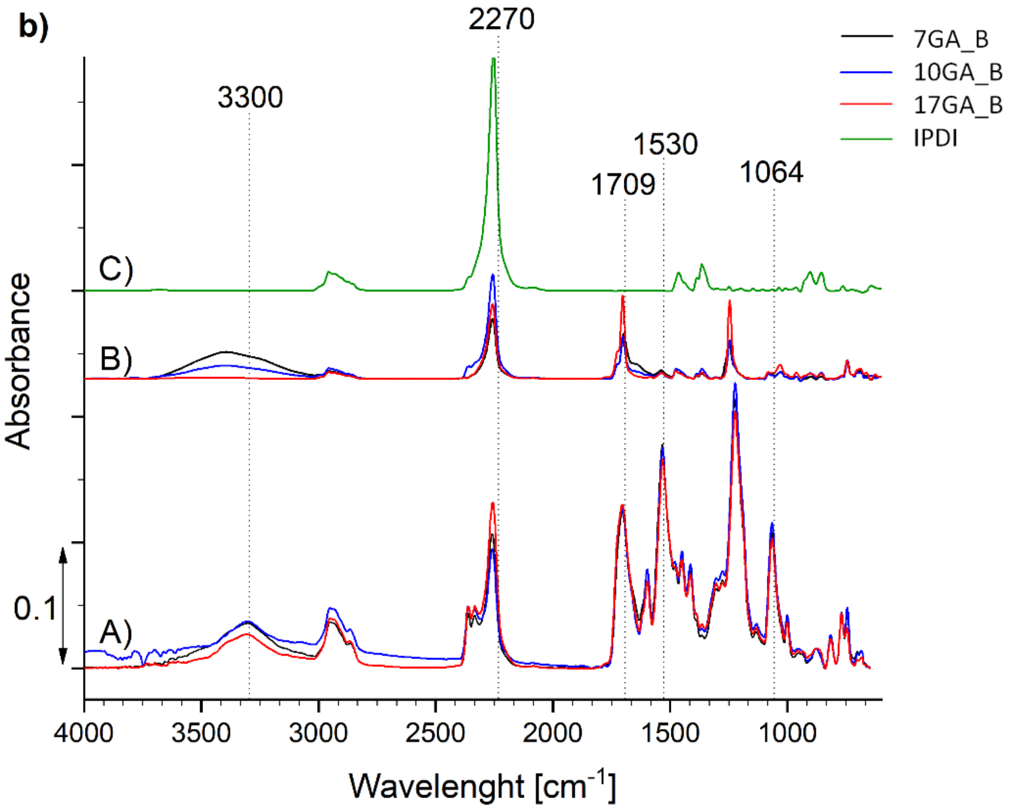

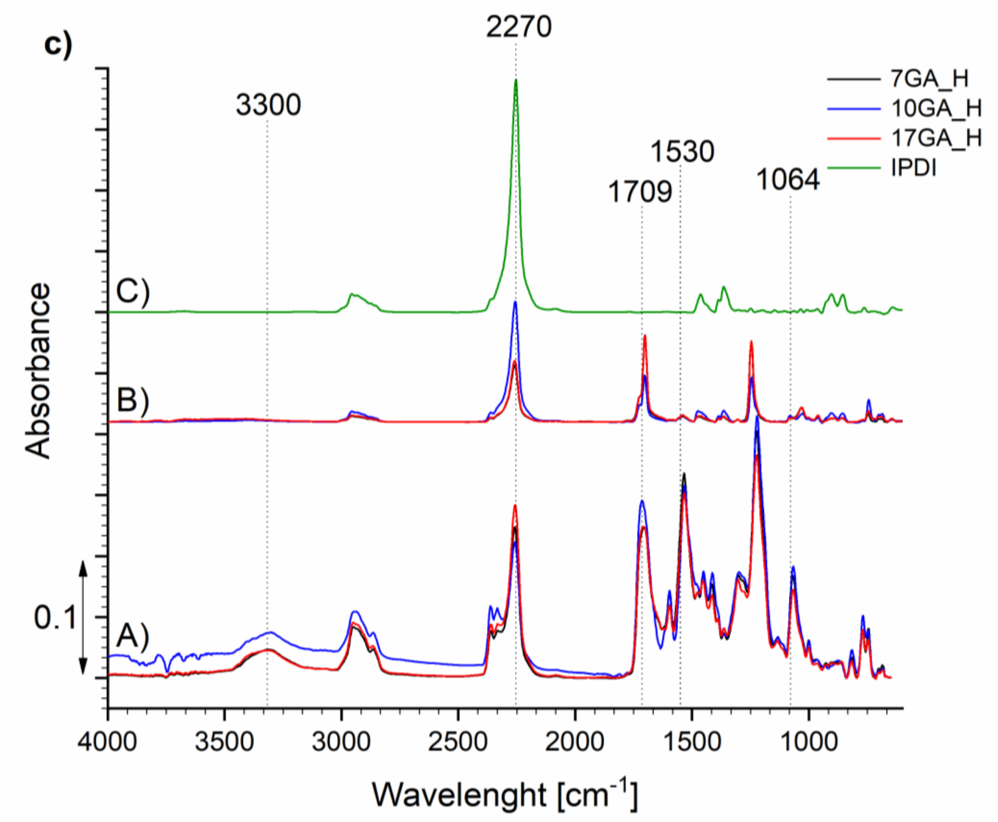

Figure S3. IR spectra of the crushed microcapsules (specters A), filtered core material (specters B) and pure IPDI (specters C) prepared from prepolymers TDIprep_G **(a)**, TDIprep_B **(b)** and TDI-prep_H prepolymers **(c)**, synthesized in the presence of 7-, 10- or 17-wt% of GA.

S4 Photo and SEM images of microcapsules after the synthesis

Photographs of the synthesized microcapsules before the incorporation in the composite coatings and the SEM images of the samples are presented in **Figure S4**. The microcapsules 10GA_B and 10GA_H formed large agglomerates, while the microcapsules 10GA_G formed a fine homogenous powder. The agglomerated microcapsules were investigated usingSEM which showed that studied microcapsules formed bonds with each other (marked with red arrows), which could be explained by leaking of the IPDI incorporated within the microcapsules. Due to this phenomenon, microcapsules 10GA_B and 10GA_H were not incorporated into the composite coatings and the research was continued with microcapsules 10GA_G.

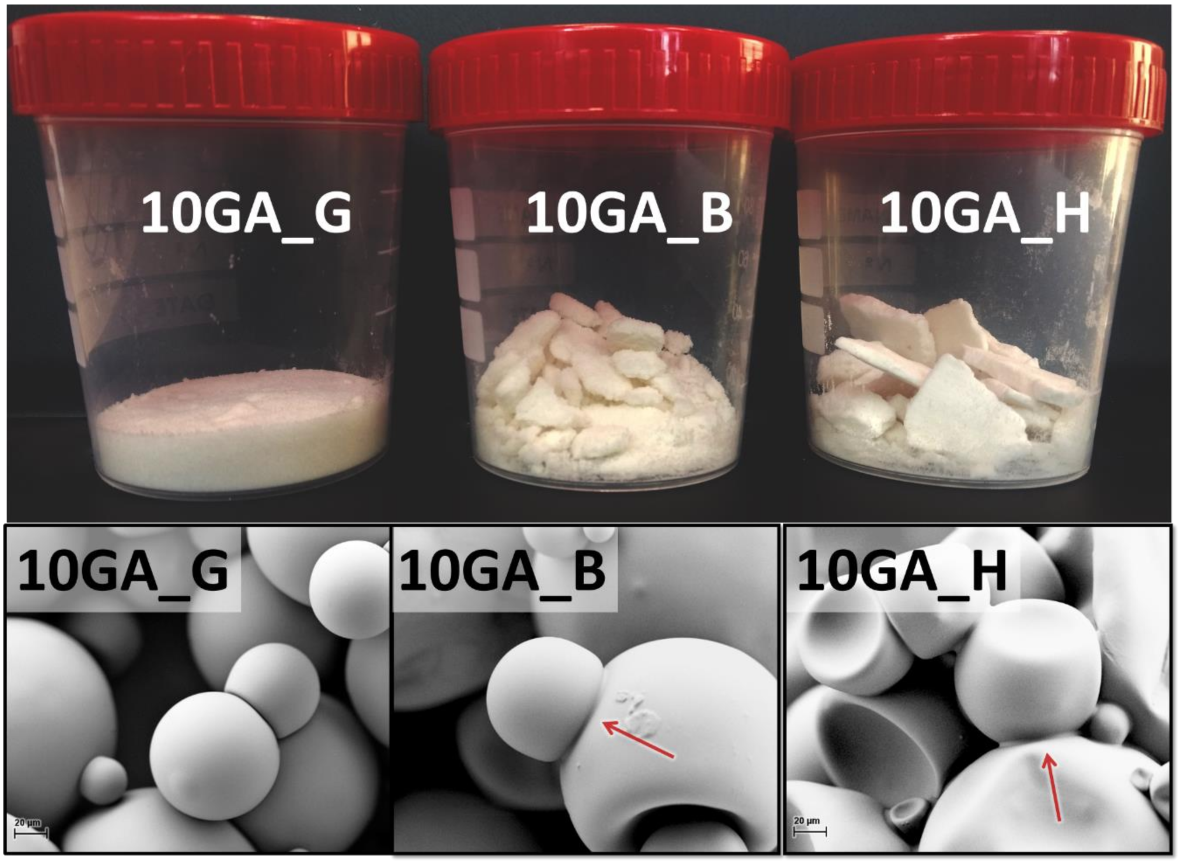

Figure S4. Photographs (upper row) and SEM images (lower row) of the microcapsules 10GA_G, 10GA_B, 10GA_H.

S5 SEM images of the applied coatings

SEM micrograph of the coating applied to metal plate is presented in **Figure S5**.

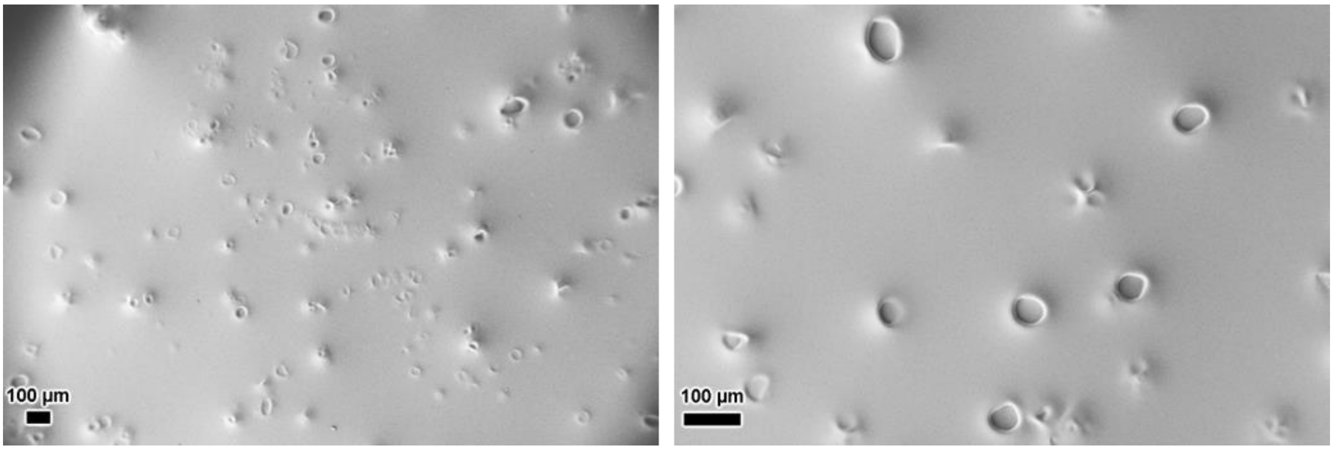

Figure S5. SEM images of the composite anticorrosion coatings applied on metal plate taken at 100 × magnification (left) and 250 × magnification (right).

The SEM images of the coated metal plates reveal that the applied composite coating is covering the entire surface of the metal plate and that the microcapsules are randomly distributed within the coating matrix. The coating does not contain any damaged areas or pores, which is beneficial for the anticorrosion protection and implyes that the coating was curred correctly.
